# Supplementary material for: Identification of Common Cancer Antigens Useful for Specific Immunotherapies to Colorectal Cancer and Liver Metastases
Source: Int J Mol Sci. 2025 Jul 31;26(15):7402. doi: 10.3390/ijms26157402 (PMC12347713; doi:10.3390/ijms26157402)
Supplement: Supplementary file 1 [file ijms-26-07402-s001.zip › 20250531_Supplementary_material.docx]

**Supporting Information**

**Figure S1.** Box plot depicting the expression scores and levels of seven common cancer antigens and HLA class I for naïve and chemotherapy groups in 60 cases of colorectal cancer liver metastases.

Sixty cases of colorectal cancer liver metastases were divided into naïve, consisting of 12 patients (20.0%) who underwent liver metastasis resection alone without prior chemotherapy, and chemotherapy, comprising 48 patients (80.0%) who received chemotherapy before liver metastasis resection, groups.

No significant differences in the expression of any of the common cancer antigens and HLA class I were observed between the groups. The median expression scores of these antigens and HLA class I in the chemotherapy group were similar to those in the naïve group.

The median expression levels for CLDN1, EphB4, LAT1, FOXM1, HSP105α, ROBO1, SPARC, and HLA class I in the chemotherapy (n=48) and naive (n=12) groups were:

CLDN1: chemotherapy 4.3 (range 0–6.0), naïve 4.3 (range 3.3–6.0)

EphB4: chemotherapy 4.0 (range 0–6.0), naïve 5.0 (range 0–6.0)

LAT1: chemotherapy 3.7 (range 0–6.0), naïve 3.7 (range 1.3–5.7)

FOXM1: chemotherapy 2.7 (range 0–6.0), naïve 2.5 (range 0–5.0)

HSP105α: chemotherapy 5.0 (range 0–6.0), naïve 4.7 (range 3.3–6.0)

ROBO1: chemotherapy 3.7 (range 0–6.0), naïve 4.2 (range 0–6.0)

SPARC: chemotherapy 2.7 (range 0–4.3), naïve 2.3 (range 1.3–5.0)

HLA class I: chemotherapy 3.7 (range 0–6.0), naïve 3.7 (range 0–5.0)

**Figure S2.** Box plot depicting the expression scores of seven common cancer antigens and HLA class I for 14 cases of primary colorectal cancer and liver metastases.

Significant differences in FOXM1 and SPARC expression scores were observed between primary colorectal cancer (n=14) and liver metastases (n=14) (p < 0.05). The median expression scores for FOXM1 and SPARC were 4.5 (range 2.7–6.0) and 5.7 (range 1.7–6.0) in primary colorectal cancer, and 2.3 (range 0.3–3.3) and 2.7 (range 0–5.0) in liver metastases, respectively. No significant difference in HLA class I expression scores was observed between primary colorectal cancer (n=14) and liver metastases (n=14) (p=0.18). The median expression score level was 4.0 (range 0–5.3) in primary colorectal cancer and 5.0 (range 1.7–6.0) in liver metastases.

The median expression levels for CLDN1, EphB4, LAT1, FOXM1, HSP105α, ROBO1, SPARC, and HLA class I about primary CRC(n=14) and CRCLM (n=14) were:

CLDN1: CRC 5.0 (range 0–6.0), CRCLM 4.3 (range 0–6.0)

EphB4: CRC 5.7 (range 0–6.0), CRCLM 5.5 (range 1.7–6.0)

LAT1: CRC 5.0 (range 3.0–6.0), CRCLM 5.2 (range 0–6.0)

FOXM1: CRC 4.5 (range 2.7–6.0), CRCLM 2.3 (range 0.3–3.3)

HSP105α: CRC 5.0 (range 0–6.0), CRCLM 5.0 (range 3.3–6.0)

ROBO1: CRC 5.0 (range 0.7–6.0), CRCLM 5.0 (range 2.7–6.0)

SPARC: CRC 5.7 (range 1.7–6.0), CRCLM 2.7 (range 0–5.0)

HLA class I: CRC 4.0 (range 0–5.3), CRCLM 5.0 (range 1.7–6.0)
