# Supplementary figures and images for: Identification of Common Cancer Antigens Useful for Specific Immunotherapies to Colorectal Cancer and Liver Metastases
Source: Int J Mol Sci. 2025 Jul 31;26(15):7402. doi: 10.3390/ijms26157402 (PMC12347713; doi:10.3390/ijms26157402)

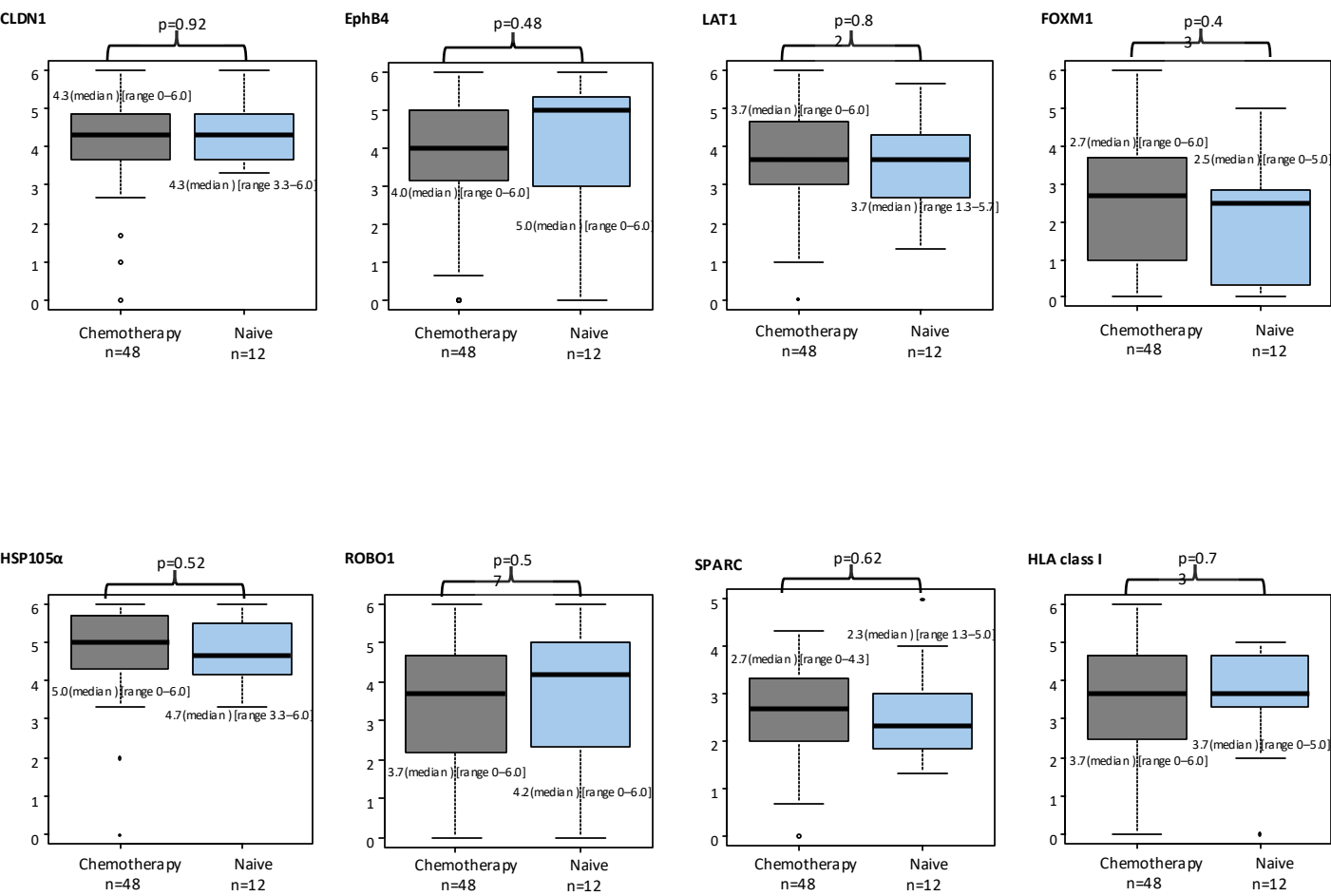

Supplementary Figure 1

Supplement: Supplementary file 1 [file ijms-26-07402-s001.zip › 20250531_Supplementary_Figure_1.pdf]

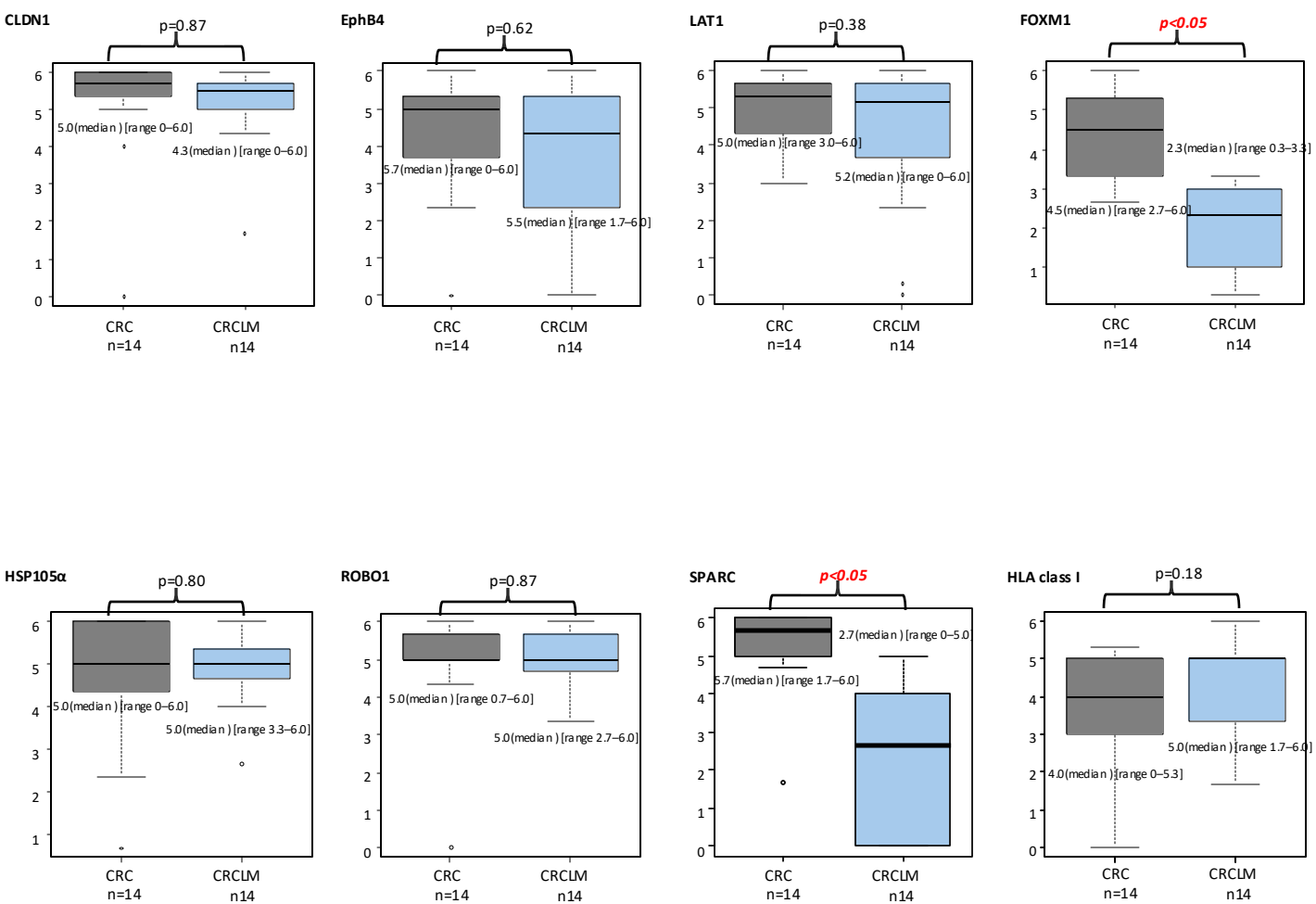

Supplementary Figure 2

Supplement: Supplementary file 1 [file ijms-26-07402-s001.zip › 20250531_Supplementary_Figure_2.pdf]
